# Effect of Dance Movement Therapy on Depression and Anxiety for Persons with Dementia: A Systematic Review

**DOI:** 10.1093/geroni/igab046.2429

**Published:** 2021-12-17

**Authors:** Ying Wang, Mandong Liu, Zhixiao Dong, Jing wu, Huan Cui, Dianjun Shen, Youyou Tan

**Affiliations:** 1 Lanzhou University, Lanzhou, Gansu, China (People's Republic); 2 University of Southern California, Hangzhou, China (People's Republic)

## Abstract

Dance movement therapy（DMT）is a physical and psychological intervention, but there was no meta-analysis of RCTs in the systematic review of DMT for dementia, and the results of RCTs were inconsistent. The study aimed to assess the effectiveness of dance movement therapy on depression and anxiety of persons with dementia in comparison to no treatment, standard care. Six electronic databases (Cochrane, PsycINFO, Web of Science, PubMed, EBSCO, CNKI) were searched through January 2021. Randomized controlled trials (RCTs) in Chinese and English language were considered, including: (1) population is women and men of all age with dementia and MCI; (2) intervention is dance movement intervention; (3) comparison is no treatment or standard care; (4) outcome is depression or anxiety. The four review authors independently reviewed studies on an abstract/title level and again after reading the full paper, and we independently evaluated methodological quality. Six randomized controlled trials were identified on depression and anxiety symptoms in persons with dementia. The target sample size was 29-842 older adults. Meta-analysis showed there were significant differences in favor of dance in depression (SMD = 1.17, 95% CI: 0.39 to 1.95, P = 0.003), but not in outcomes of anxiety. Trials of high methodological quality, large sample sizes, and clarity in the way the intervention is put together and delivered are needed to assess whether dance movement therapy is an effective intervention on depression and anxiety for persons with dementia.

